# The mTOR signaling pathway in cardiac aging

**DOI:** 10.20517/jca.2023.10

**Published:** 2023-05-04

**Authors:** Dao-Fu Dai, Ping Kang, Hua Bai

**Affiliations:** 1 Department of Pathology, University of Iowa, Iowa City, IA 52242, USA.; 2 Department of Genetics and Cell Biology, Iowa State University, Ames, IA 50011, USA.

**Keywords:** mTOR, aging, cardiac aging, heart failure, rapamycin, caloric restriction

## Abstract

The mammalian target of rapamycin (mTOR) is one of the most important signaling pathways that regulate nutrient sensing, cell growth, metabolism, and aging. The mTOR pathway, particularly mTOR complex 1 (mTORC1), has been shown to control aging, lifespan, and healthspan through the regulation of protein synthesis, autophagy, mitochondrial function, and metabolic health. The mTOR pathway also plays critical roles in the heart, from cardiac development, growth and maturation, and maintenance of cardiac homeostasis. Hyperactivation of mTORC1 signaling is well documented in aging and many age-related pathologies, including age-related cardiac dysfunction and heart failure. Suppression of mTORC1 by calorie restriction or rapamycin not only extends lifespan but also restores youthful phenotypes in the heart. In this article, we review model organisms of cardiac aging and highlight recent advances in the impact of the mTORC1 pathway on organismal and cardiac aging, particularly in *Drosophila* and mice. We focus on the downstream signaling pathways S6 kinase and 4EBP1, which regulates protein synthesis, as well as ULK1 and its related pathway that regulates autophagy. The interaction with mTOR complex 2 (mTORC2) and its potential role in cardiac aging are also discussed.

## INTRODUCTION

The target of rapamycin (TOR) was originally identified in yeast as the cellular target of the immunosuppressant and anticancer drug known as rapamycin^[1]^. In mammals, two mTOR complexes with distinct structures and functions are found - the mTOR complex 1 (mTORC1) and the mTOR complex 2 (mTORC2). The mTORC1 consists of mTOR, Raptor (regulatory-associated protein of mTOR), and mammalian LST8 homolog (mLst8). The mTORC1 activity is modulated by several additional components, including DEPTOR, the DEP domain containing mTOR interacting protein, and the PRAS40. The mTORC2 contains mTOR, the rapamycin-insensitive companion of mTOR (Rictor), mLST8, and the mammalian stress-activated protein kinase-interacting 1 (mSin1). Further, the additional components of mTORC2 include DEPTOR and the protein observed with Rictor 1 and 2 (also known as Protor1/2)^[2,3]^ (see review^[4]^ for detail).

**Figure 1.**
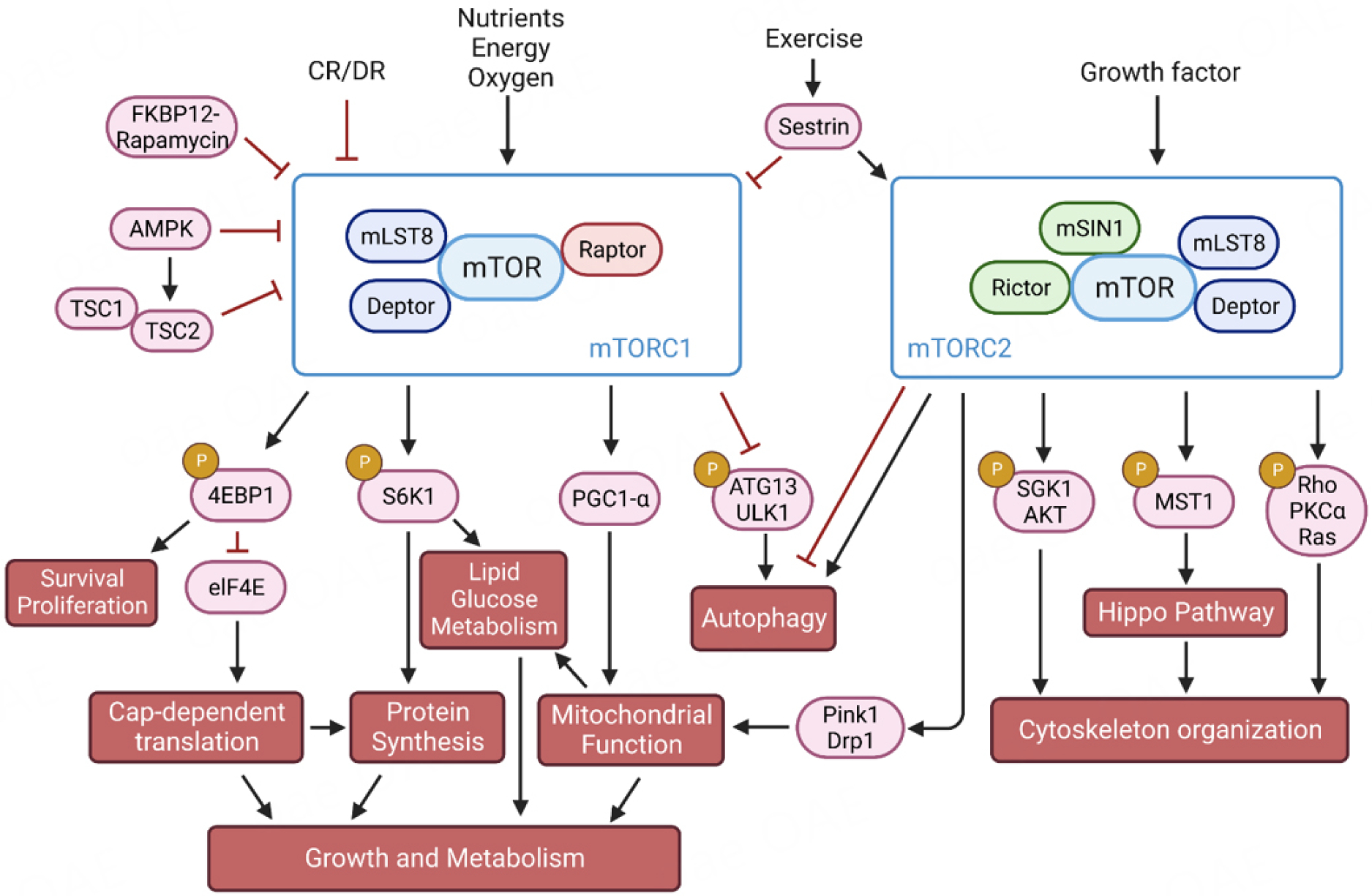
mTORC1 and mTORC2 signaling in the regulation of protein synthesis, lipid and glucose metabolism, mitochondrial function, autophagy, and cytoskeleton organization.

The mTORC1 activity is finely tuned by several environmental cues, including the levels of intracellular nutrients and energy, growth factors as well as other cellular stresses [Figure 1]. The current review will focus on downstream pathways regulated by mTOR and will briefly discuss the upstream regulators of mTOR [Figure 1].

## UPSTREAM REGULATORS OF MTOR

mTORC1 is activated by nutrients, growth factors and mitogen-dependent signaling. Many of these signals inhibit the Tuberous Sclerosis Complex (TSC, a key negative regulator of mTORC1), which then activate the small GTPase Rheb^[5]^. Conversely, mTORC1 is inactivated by nutrient deprivation. On the one hand, upon depletion in cellular energy, adenosine monophosphate (AMP)-activated protein kinase (AMPK) is activated to block mTORC1 activities via the phosphorylation of either TSC2 or Raptor^[6,7]^. On the other hand, glucose deprivation inhibits mTORC1 via Rag GTPases in an AMPK-independent manner^[8,9]^. These are consistent with the essential role of mTORC1 in nutrient sensing and growth promotion^[10]^ [Figure 1].

Amino acids are major nutrients activating mTORC1. During aging, mTORC1 is activated by food intake, particularly amino acid availability. Breakdown of dietary proteins from food intake increases serum amino acid levels. Amino acid stimulates the Rag GTPases to facilitate the binding to Raptor and activation of mTORC1^[11,12]^. GATOR1 and GATOR2 complexes have been reported as amino acid sensors and upstream activators of mTORC1^[13]^. Branched-chain amino acids (BCAA, including isoleucine, leucine and valine) are potent activators of mTORC1 that increase protein synthesis, as well as regulate lipid and glucose metabolisms. In addition, a high concentration of BCAA induces mitochondrial dysfunction and ROS, promotes the activation of NF-κB, and increases the expression of cytokines and adhesion molecules in peripheral blood mononuclear cells^[14]^. The effect of BCAA in increasing oxidative stress and inflammation is mediated through the activation of mTORC1^[14]^. This links many signaling pathways that have been implicated in the aging process, including ROS, inflammation and mTOR.

Besides nutrient sensing, mTORC1 is involved in the regulation of hypoxia and DNA damage responses. For example, mTORC1 is inhibited by AMPK and REDD1 under hypoxia^[15]^, while DNA damage inhibits mTORC1 through p53 activation^[16]^. Interestingly, resistance exercise inhibits mTORC1 via AMPK and Sestrins^[17]^. Sestrins are a family of evolutionarily conserved exercise-inducible proteins that mediate the metabolic benefits of exercise through mTORC1 inhibition and mTORC2 activation^[18,19]^. Molecular and genetic evidence suggests that Sestrins are induced by the FOXO transcription factor, while Sestrins inhibit mTORC1 via activation of AMPK and TSC2^[20,21]^. The upstream regulators of mTOR signaling are discussed in further detail in the previous review^[10]^.

mTORC2 can also be activated by growth factor pathways, in particular insulin/PI3K signaling [Figure 1]. Upon the activation of PI3K signaling, PtdIns(3,4,5)P3 binds to the pleckstrin homology (PH) domain of mTORC2 subunit SIN1, which releases the autoinhibition on the mTOR kinase domain and triggers mTORC2 activation^[22]^. The mTORC2 activity can also be influenced by mTORC1. Two phosphoproteomic studies identify that mTORC1 phosphorylates and activates Grb10 to negatively control insulin/IGF-1 signaling, the upstream effector of mTORC2^[23]^. In addition, mTORC1 target S6K1 inhibits mTORC2 through the phosphorylation of insulin receptor substrate 1 (IRS1)^[24,25]^

## DOWNSTREAM SIGNALING PATHWAYS

### Regulation of protein synthesis through S6 Kinase and 4EBP1

Overall, mTORC1 activation increases the expression of genes related to growth and metabolism^[26]^. However, it decreases the expression of genes involved in stress adaptation^[27]^. To support growth, mTORC1 plays a major role in controlling protein synthesis. mTORC1 activation promotes protein translation through both S6 kinase 1 (S6K1) and eukaryotic initiation factor 4E (eIF4E) binding proteins (4E-BP)^[26]^ [Figure 1]. S6K1 upregulates genes for ribosomal proteins, ribosome biogenesis factors, and tRNAs, leading to ribosome biogenesis^[28–31]^ and promoting protein synthesis. Ribosomal protein S6 is the substrate of S6K1 that has been used as a marker of mTORC1 activity^[32]^. When phosphorylated and activated by mTORC1, S6K1 phosphorylates programmed cell death protein 4 (PDCD4), which is then targeted for degradation^[33]^. The PDCD4 functions to inhibit protein translation by blocking eIF4A helicase that controls ribosome biogenesis. The ability of mTORC1 to phosphorylate and activate S6K1 is modulated by the alterations of intracellular ATP levels^[34]^. S6K1 regulates cell size, growth, and metabolism^[35]^. The latter is modulated by adenosine monophosphate (AMP)-activated protein kinase (AMPK)^[36]^. This interaction between mTOR and AMPK links the major nutrient pathway and energy sensor signaling pathways within the cells.

The mTORC1 also targets 4E-BP1 through phosphorylation and releases the latter from eiF4E, thereby derepressing the cap-dependent initiation of translation^[37]^. The eukaryotic initiation factor - 4F (eIF4F) complex - regulates the association between mRNA cap-binding protein - eIF4E - and scaffold protein - eIF4G, thus mediating growth-dependent protein synthesis^[38]^. Consequently, this process leads to mRNA circularization and an increased translation rate^[38]^.

### Inhibition of autophagy

Autophagy is an essential cellular homeostasis process responsible for the degradation and recycling of damaged macromolecules and organelles^[39]^. This essential cellular process is also modulated by mTOR. Autophagy is activated or inhibited in response to a variety of upstream signaling, such as nutrient signaling (mTOR, insulin, AMPK), Ras/PKA pathway, ER stress, hypoxia, oxidative stress and pathogen infection^[40]^. Autophagic activity declines in aged tissues^[41]^, including the hypothalamus^[42]^, liver^[43]^, skeletal muscle^[44]^, and heart^[45,46]^. Studies in model organisms demonstrate a direct link between autophagy and longevity control since genetic disruption of autophagy genes blocks the lifespan extension by insulin-like signaling mutants, dietary restriction, and rapamycin treatment^[47–49]^. Furthermore, suppression of autophagy can lead to cardiomyopathy^[46]^, while activation of autophagy by disruption of beclin1 significantly increases lifespan and diminishes age-related cardiac hypertrophy and fibrosis^[50]^.

Autophagic activity can be enhanced by fasting or rapamycin, both of which are mediated by mTORC1 inhibition^[51,52]^. Mechanistically, mTORC1 inhibits autophagy by the phosphorylation of Unc-51 Like Autophagy Activating Kinase 1 (ULK1), ATG13^[53]^, and transcription factor EB (TFEB)^[54,55]^ [Figure 1]. Inhibition of mTOR by rapamycin (RP) treatment results in the dephosphorylation of ULK1, which then initiates autophagosome formation^[53]^. In addition, the mTORC1 also phosphorylates TFEB, a master transcriptional regulator of autophagy and lysosome genes. The phosphorylation of TFEB prevents its nuclear localization and reduces autophagy through transcriptional regulation^[54,55]^.

The age-dependent decline of autophagy has been linked to elevated mTOR activity observed in many, but not all, aging tissues^[56,57]^. The decline of autophagic activity in many aged tissues can be restored by mTOR inhibition^[39]^. The critical role of mTOR regulation of autophagy in aging is supported by the finding that the lifespan extension induced via mTOR inhibition can be attenuated by knocking down autophagy genes, such as *Ulk1*^[48,58]^, *hlh-30/Tfeb*^[59]^, and *Atg5*^[49]^. These studies show that the pro-longevity effect of mTOR inhibition is at least partly mediated by an autophagy-dependent mechanism. Furthermore, TGFβ/activin-regulated inhibition of mTORC2 has been recently shown as another mechanism for the age-dependent decline of autophagic activity in *Drosophila* cardiomyocytes^[45]^. Either knockdown of TGFβ/activin ligand *daw* or overexpression of mTORC2 subunit *Rictor* rescue autophagy in aged cardiomyocytes and preserved cardiac function with age^[45]^. However, the negative regulation of mTORC2 on autophagy has been previously reported. AKT inhibits autophagy via direct phosphorylation of Beclin-1, while overexpression of Rictor attenuates MIR211-induced autophagy in HeLa cells^[60,61]^. Besides bulk autophagy, mTOR signaling has also been linked to mitophagy regulation under oxidative stress^[62]^. However, how mTOR-regulated mitophagy contributes to cardiac aging remains to be further determined.

### Regulation of lipid metabolism

Metabolic syndrome is well-known to increase the risk of cardiovascular diseases. Excessive dietary fat consumption or pathogenic liver lipogenesis promotes cardiac lipotoxicity in cardiomyocytes, which may lead to cardiomyopathy^[63]^. In animal models fed with a high-fat diet, fatty acid oxidation is upregulated, and mitochondrial energetics is reduced, predisposing to cardiomyopathy resembling those found in diabetes or cardiometabolic syndrome. The cardiac dysfunction may manifest as either reduced or preserved left ventricular ejection fraction (systolic function), and left ventricular diastolic function is commonly impaired^[64–68]^. Thus, maintaining the metabolic health of the heart is crucial for its normal function.

Besides its key role in amino acid sensing and protein synthesis, mTOR also regulates lipid metabolism (see reviews^[69–71]^). Genetic manipulations of either mTORC1 or mTORC2 signaling components can lead to aberrant lipid metabolism. Hyperactivation of mTORC1 via liver-specific *TSC1* deletion blocks ketogenesis in fasted mice^[72]^. Deletion of *TSC2* also leads to enhanced adipogenesis^[73]^. Additionally, mice with *S6K1* or *RAPTOR* deletion show reduced adipogenesis and are resistant to high-fat diet-mediated weight gain^[74,75]^. It is known that mTORC1 regulates lipogenesis through transcriptional and post-transcriptional regulation of SREBP transcription factors^[76,77]^. Recently, mTORC1 has been found to regulate *de novo* lipogenesis via histone demethylase JMJD1C^[78]^ and splicing regulator SRPK2^[79]^. Similar to mTORC1, mTORC2 also positively controls lipogenesis. The adipocytes with *Rictor* knock-out mice show elevated basal lipolysis, while liver-specific *Rictor* knock-out blocks insulin-induced hepatic lipogenesis^[80,81]^, likely due to the dysregulated transcription of the key lipogenic genes.

A high-fat diet is a major cause of metabolic syndrome that predisposes to cardiovascular diseases. In *Drosophila*, high-fat diet-induced cardiomyopathy and excess fat accumulation can be attenuated by loss-of-function mutations of TOR^[67]^. In this context, both SREBP and PGC-1α are shown to be the downstream effectors in mediating TOR-regulated cardiac lipotoxicity^[68]^. In mice fed with a high-fat diet, cardiomyocyte autophagy is inhibited because of mTORC1 activation, while rapamycin or Rheb inactivation can restore autophagic activity and protect hearts from ischemia injury^[82]^. Consistent with this, rapamycin (RP) administration can also rescue obesity-associated cardiac hypertrophy, contractile dysfunction and fractional shortening in obese mouse models^[83,84]^. Further, mTORC1 inactivation by overexpressing PRAS40 prevents the development of diabetic cardiomyopathy and improves hepatic insulin sensitivity^[85]^.

It has been shown that the aging heart is accompanied by a significant alteration in lipid metabolism. The old heart exhibits a decrease in fatty acid oxidation and an increase in glycolysis^[86]^. The reduced fatty acid oxidation in old hearts could lead to fat accumulation and, eventually, lipotoxicity and cardiomyopathy during aging. Recently, we found that a high-fat diet activates mTORC2 in *Drosophila* hearts, while loss of Rictor exaggerates cardiac contractile defects under high-fat diet treatment^[87]^. The cardiac protective role of mTORC2 might be due to its role in Drp1-mediated mitochondrial fission^[87]^.

## THE MTOR REGULATION OF ORGANISMAL AGING

In the aging process, the mTORC1 pathway is considered a major signaling mechanism. This significant role of mTOR signaling in aging was first demonstrated in *C. elegans*. Decreased expression of ceTOR (previously referred to as *let-363,* a homolog of mTOR) or *daf-15* (a homolog of Raptor) aided in the life span extension of *C. elegans*^[88,89]^. mTOR contribution in this lifespan is further validated by the fact that the suppression of TOR signaling extended the lifespan of *Saccharomyces* (budding yeast)^[90]^, *Drosophila*^[91]^, and mice^[92,93]^. The mechanisms underlying the lifespan extension of mTORC1 inhibition may include suppression of protein synthesis (via eIF4E/4E-BP1 and *S6K*), enhancement of proteostasis (such as via ULK1 regulation of autophagy) and attenuation of oxidative stress^[10]^. In *C. elegans*, depletion of the eIF4F cap-binding complex component protein facilitates the lifespan extension process^[94]^. In mice, loss of the mTORC1 substrate S6 kinase (S6K1) extended lifespan and ameliorated several aging phenotypes in the musculoskeletal, immune, and metabolic systems^[95]^. In this context, the gene expression analysis of these mice revealed alterations resembling those observed in response to caloric restriction or AMPK activation^[95]^.

It is pertinent to mention that Caloric Restriction (CR) is widely acknowledged as the most reproducible intervention for lifespan extension in numerous organisms, such as yeast, worm, fruit fly, and mice^[96]^. CR also extends the healthspan through the amelioration of various age-related diseases, including cardiovascular diseases^[97]^, cancer^[98]^, neurodegenerative diseases, retinal aging and macular degeneration^[99,100]^, and age-dependent sensorineural hearing loss^[101]^. In non-human primates, moderate CR attenuated age-related pathological conditions, including diabetes, cancer, neurodegeneration, and cardiovascular diseases^[102]^. As briefly discussed above, hyperphagia with increased caloric intake resulted in lifespan shortening in various model organisms.

The mTORC1 is a master regulator of nutrient sensing^[10]^. Moreover, several studies support that mTORC1 mediates the beneficial anti-aging effects of CR. The overlapping mechanism of mTORC1 inhibition and CR is reinforced by the absence of additional life span extension by CR in yeast^[90]^, worms^[103]^, or flies^[91]^ with decreased mTORC1 signaling. Downstream of mTORC1, translational modulation through 4E-BP is shown to mediate the protective effect of Dietary Restriction (DR)^[37]^ in *Drosophila.* In this context, DR resulted in differential loading of mRNA onto ribosomes that later led to the preferential translation of mRNA of various mitochondrial proteins. This included the components of mitochondrial electron transport chains mediated by the upregulation of 4E-BP. The enhancement of mitochondrial activity and lifespan extension of *Drosophila* were dependent on 4E-BP^[37]^. Another study reported that muscle-specific overexpression of 4E-BP in *Drosophila* contributed to lifespan extension and proteostasis improvement^[44]^. Even though no apparent proof regarding the mediation of S6K1 on the beneficial effects of CR (as demonstrated for 4E-BP in *Drosophila* models) has surfaced, the contribution of S6K1 in the longevity is reflected in both the lifespan and healthspan extension in S6K1 knockout mice and the gene expression patterns that are similar to the CR effects^[95]^.

The role of rapamycin (RP) as an inhibitor of mTORC1 highlights its ability to prolong both healthspan and lifespan in mammals and many model organisms that have been studied^[49,104,105]^. By inhibiting mTORC1, RP treatment led to several outcomes: global protein synthesis suppression, cell growth inhibition, activation of stress response pathway and autophagy^[106]^. Furthermore, mTOR complex I inhibition diminishes the energetic burden of protein translation, causes a reduction in oxidative stress, pauses the accumulation of deleterious metabolic by-products, and contributes to the enhancement of the autophagic removal of damaged macromolecules. Consequently, it leads to the improvement of cellular function^[37,106]^.

It has been observed that the initiation of rapamycin in the later stages of life extended lifespan of genetically heterogeneous mice. It needs to be emphasized that the lifespan extension effect of RP has been reproducible by multiple independent observations reported by Harrison *et al*.^[104]^. This multi-institutional study also found gender and dose effects of RP in lifespan extension. It was revealed that each dose of RP increased the female lifespan more compared to the male counterparts^[104,107]^. Researchers attribute this gender difference to the sex difference in RP metabolism and blood levels^[107]^. Although RP was regarded to be a CR mimetic, it was not fully supported by extensive metabolomic and transcriptomic studies in mammalian white adipose tissue, liver, and blood. RP and CR did show some overlapping effects, yet there were distinctively unique and non-overlapping effects of CR and RP reflected in these tissues^[108–110]^. Indeed, it has been reported that subacute CR and RP discordantly altered mouse liver proteome homeostasis and reversed aging effects^[111]^. Further, RP demonstrates various endocrine and metabolic modifications in mice in a sex and dose-dependent manner, different from the CR effect^[107]^. However, both RP and CR attenuate protein oxidative harms and enhances overall protein quality^[111]^. Taken together, RP shares some overlapping mechanisms with CR, yet it also has some distinctive effects in multiple organ systems at the levels of proteomes and metabolomes.

## MODEL ORGANISMS FOR CARDIAC AGING STUDIES

### Drosophila cardiac aging model

*Drosophila melanogaster*, the fruit fly, has been widely used as an excellent genetic model to understand the mechanisms of cardiac aging. *Drosophila* not only provides a tractable genetic system, but the molecular signaling pathways governing heart development and aging are also highly conserved between flies and vertebrates^[112–115]^. The adult *Drosophila* heart is a linear tube consisting of 40–50 pairs of mature cardiomyocytes^[116]^. The first transcription factor (Tinman) that controls animal heart development was discovered in *Drosophila*^[117]^, and this factor is highly conserved in vertebrates^[118,119]^. In addition, *Drosophila* hearts display an age-dependent decline in structure and function, similar to that found in mammalian hearts. For example, aged *Drosophila* hearts exhibit diastolic dysfunction, decreased fractional shortening, increased stiffness, increased collagen deposition, and impaired calcium handling, *etc*.^[113]^. These resemblances to mammalian cardiac aging make *Drosophila* an amenable genetic model in dissecting the mechanisms underlying cardiac aging.

The role of *Drosophila* as a model of cardiac aging is further supported by advances in innovative imaging analysis. For example, the Semi-automated Optical Heartbeat Analysis (SOHA) was developed to monitor fly heart wall movement using a high-speed digital camera and movement analysis algorithms^[120]^. The SOHA method offers high sensitivity in detecting subtle contractile defects, including changes in fractional shortening and cardiac arrhythmia^[121]^. Fluorescence-based optical heartbeat analysis on intact animals was developed by expressing fluorescence proteins in the hearts. Although the original method is limited by speed and resolution^[122,123]^, a novel heart enhancer R94C02 has greatly improved the imaging resolution^[124]^. Optical Coherence Tomography (OCT) has been used as a noninvasive imaging technique to analyze the heart parameters from unanesthetized intact flies^[125]^. Atomic force microscopy (AFM), capable of evaluating the cardiomyocyte mechanical properties^[126]^, has been applied to study age-related changes in cardiac stiffness^[127,128]^ and cardiac remodeling of Troponin-T mutants in *Drosophila*. All of the above tools make the *Drosophila* heart system a powerful model for dissecting the determinants of cardiac aging.

Related to cardiac aging, long-lived *Drosophila* mutants in the insulin/insulin-like growth factor (IIS) signaling, such as InR and chico mutants, show preserved cardiac function with age^[129]^. The effects of IIS in cardiac aging are cell-autonomous, as cardiac-specific overexpression of *dPTEN* or *dFOXO* prevents age-related decline in cardiac performance^[129]^. The roles of TOR in *Drosophila* cardiac aging are demonstrated by genetic manipulation of its downstream target d4eBP and dS6K^[130]^. Both *dS6K* mutants and heart-specific *d4eBP* overexpression prevents the age-related decline of cardiac function and improves cardiac stress resistance, whereas heart-specific *dTOR* overexpression increases stress-induced heart failure at a young age^[130]^. Hyperactivation of TOR by Sestrin (dSesn) mutations results in dilated hearts and increased arrhythmia^[20]^. Sestrins belong to a family of exercise-inducible proteins and are key regulators in meditating exercise benefits in flies, including age-related cardiac protection^[18–20]^. Mechanistically, gene expression analysis of aging fly hearts showed upregulation of metalloproteases, genes involved in DNA replication and repair, and downregulation of genes in the mitochondrial matrix and carbohydrate metabolism^[131]^. A comparative meta-analysis with published rodent cardiac transcriptomics displays similarities in many age-related alterations between fly and rodent hearts, including extra-cellular matrix remodeling, mitochondrial metabolism, protein handling, and contractile functions^[131]^.

Another recent study from our laboratory used *Drosophila* to uncover a novel role of TGF-β/activin signaling in regulating cardiac aging. We demonstrate that activin-mediated inhibition of mTORC2 is a novel mechanism responsible for the age-dependent decline of autophagy and cardiac health in *Drosophila*^[45]^. In summary, the sophisticated genetic tools and advanced imaging techniques will continue driving future discoveries using *Drosophila* as a model in cardiac aging research.

### Murine models of cardiac aging

For studying cardiac aging, mice are considered the most valuable models. The extensive use of genetically modified mice offers invaluable tools to clarify the molecular mechanisms of cardiac aging. Since many of the laboratory mouse strains do not develop spontaneous hypertension, diabetes, or abnormal cholesterol with aging^[132]^, the age-dependent changes in cardiac structures, function, or molecular compositions likely reflect intrinsic cardiac aging^[133]^. In the absence of the above-confounding factors, mice demonstrate cardiac aging changes that closely resemble the alterations in human cardiac aging (as reported in the Framingham Heart Study and Baltimore Longitudinal Study on Aging)^[133–135]^. Our longitudinal echocardiography study on a mouse longevity cohort^[133]^ demonstrated an age-dependent increase in the left ventricular mass index (indicating the left ventricular hypertrophy) as well as an age-dependent decline in diastolic function measured by the ratio of early-to-late Tissue Doppler measurement of diastolic mitral annular velocity (Ea/Aa) and dilation of the left atrium. Only a modest reduction of systolic function from the young to the oldest group was observed^[136]^. These findings in C57Bl6/J mice were also confirmed in mice from other strain backgrounds, including C3H, BalbC, and hybrid strains (unpublished data). Histological examinations of old mouse hearts demonstrate increased interstitial fibrosis, cardiomyocyte hypertrophy or increased myocardial fiber size, and increased variation in myocyte fiber size, occasional cytoplasmic vacuolation, collapse of sarcomeres, mineralization, arteriosclerosis and arteriolosclerosis^[137]^, increased cardiomyocyte apoptosis^[138]^ and increased deposition of amyloid^[139,140]^. In humans, senile amyloidosis composed of wild-type transthyretin-derived amyloid fibrils accumulates in the heart of elderly patients. If the accumulation of amyloid is extensive, it could lead to cardiac hypertrophy, diastolic dysfunction and progressive heart failure^[141]^. The roles of mTORC1 in murine cardiac aging have been confirmed in many studies using caloric restriction or rapamycin^[97,142]^. We previously showed that short-term caloric restriction or rapamycin reversed age-dependent LV hypertrophy and ameliorated diastolic dysfunction in old mice, in conjunction with better preservation of mitochondrial proteome^[97]^. These mitochondrial-protective beneficial effects of mTORC1 inhibition are consistent with our prior observations that protecting mitochondria by mitochondrial targeted antioxidants attenuates heart failure in various mouse models^[143–146]^. Mice with genetic disruption of mTORC1 components have been extensively studied in the context of cardiac hypertrophy, heart failure and cardiomyopathy (see below).

In addition to mouse and rat models, the naked mole-rat (NMR) is a unique rodent about the size of mice (~40 g) with extraordinary longevity (> 37 years). Potential mechanisms underlying the longevity of NMR include: (1) high levels of autophagy throughout the majority of their lifespan^[147]^(mTOR activation suppresses autophagy); (2) increased translational fidelity because of their unique 28S ribosomal RNA cleavage pattern^[148]^; (3) increased resistance to hypoxia and oxidative stress^[149]^; and (4) dampened inflammatory response^[150]^. Unlike mice that manifest several phenotypes of human cardiac aging, NMR maintains cardiac function and functional reserve capacity until late in life (~34 years), with less LV hypertrophy and less premature beat arrhythmia, and better-preserved diastolic function^[151]^.

### Large mammals in the study of cardiac aging

According to a study conducted on ~9,000 dogs of various breeds, the most common cause of death were cardiovascular diseases^[152]^. Chronic degenerative valvular heart diseases were the most prominent, whereby they caused death in ~75% of dogs (16+ years old)^[153]^. Other common cardiovascular diseases in aged dogs include dilated cardiomyopathy, amyloidosis, lipofuscinosis, and sick sinus syndrome. Cardiac aging in dogs is characterized by decreased contractility, impaired relaxation, and increased systolic and diastolic stiffness^[154,155]^. Age-related cardiac hypertrophy, increased myocardial stiffness, and reduced responsiveness to β-adrenergic stimulation are associated with progressive loss of cardiac reserve, thereby predisposing to the development of heart disease in aged dogs^[156]^.

Dog Aging Project is an NIH-sponsored multi-institutional long-term longitudinal study related to aging in companion dogs^[157]^. The purpose of initiating this project was to gain an understanding of the ways genes, lifestyle, and environment influence aging. It is expected that the research would facilitate the extension of healthspan in both people and pets. A pilot short-term rapamycin intervention trial of the project was conducted on 24 companion dogs. The results revealed that a 10-week-long rapamycin treatment demonstrated a trend of improved diastolic and systolic functions in middle-aged companion dogs^[158]^. This pilot study is consistent with the reports that rapamycin initiated at middle to old age in mice can rejuvenate several cardiac aging phenotypes^[97,159]^ and further reinforces the critical role of mTORC1 in dog cardiac aging. This ongoing study is currently recruiting more companion dogs to increase the sample size.

Similarly, primate models are indispensable to aging studies as they share similarities in complex physiological traits and demonstrate phylogenetic closeness to humans. Like the findings in humans, cardiovascular diseases are the most common cause of death in captive great apes^[160]^. The prevalence of cardiovascular diseases in primates increases in old age as well. This was proven by the longitudinal aging study conducted at the National Institute of Aging, whereby the researchers used an aging cohort of rhesus monkeys (*Macaca mulatta*). The report revealed that several cardiac aging changes in the rhesus monkeys resemble human cardiac aging, including degenerative calcifications of aortic and mitral valves, loss of cardiomyocytes, cardiomyocyte hypertrophy, accumulation of lipofuscin, and amplified interstitial fibrosis. In addition, an increased incidence of myocardial infarction, heart failure, and myocardial inflammation was observed^[161–164]^. In a 20-year longitudinal CR study initiated in adult rhesus monkeys, moderate CR has been shown to decrease the incidence of aging-related deaths and delay the onset of many age-associated pathologies^[102]^. In this study, CR reduced the incidence of diabetes (and improved insulin sensitivity), cancer and brain atrophy, and cardiovascular disease. The incidence of cardiovascular diseases, including valvular endocardiosis, cardiomyopathy, and myocardial fibrosis, was reduced by 50% in the CR group. Since mTORC1 is a major pathway suppressed by CR, these findings emphasize that mTORC1 also plays important roles in primate cardiovascular aging. Besides the rhesus monkey, the common marmoset (*Callithrix jacchus*) is the shortest-lived anthropoid primate (average lifespan: 5–7 years). They develop several aging pathologies resembling those of human beings, including cancer, amyloidosis, diabetes, and chronic kidney diseases. Histological examinations showed increased myocardial fibrosis and some features of cardiomyopathy in old marmosets over 10 years of age^[165,166]^.

A recent comparative study in multiple species of mammals reported that cardiac protein abundance of mTOR, Raptor, and PRAS40 and the phosphorylation of PRAS40^Thr246^ are inversely correlated with the maximal lifespans of mammals^[167]^. At chronological ages of 15–30% of their maximal lifespan, long-lived mammals (horse, 46 years; cow, 30 years; pig, 27 years) tend to have lower mTORC1 than the shorter-lived mammals (mice, rats, guinea pigs, gerbils and rabbits). This correlative multi-species study of mTORC1 signals in the hearts also supports the essential role of mTOR in cardiac aging.

## MTORC1 IN CARDIAC AGING AND HEART FAILURE

Studies reveal that *TOR* plays a critical role in *Drosophila* cardiac aging^[168]^. Downstream of *dTOR* signal, *d4eBP* overexpression is adequate to protect cardiac function against age-related decline while enhancing cardiac stress resistance^[130]^. Conversely, *d4eBP* null mutant flies exhibit an early increase in stress-induced failure rate. Similarly, upregulation of *dEif4e* (which is inhibited by *d4eBP*) in the heart mimics the age-related increase in cardiac arrhythmias. Another effector of *dTOR* and insulin signaling, *dS6K*, may influence cardiac aging non-autonomously through its activity in the insulin-producing cells (systemic/metabolic benefit), but not directly in the myocardial tissue. This is because myocardial dnS6K inflicts no significant effect on cardiac function, like the neutral effect of mouse S6K1/S6K2 double knock-out in response to cardiac hypertrophy stimuli^[169]^.

The mTORC1 hyperactivation leads to pathological growth and cardiac dysfunction in response to pressure overload in rodents. This mTORC1 activation induces phosphorylation of p70s6K and 4EBP1, thus stimulating ribosome biogenesis and protein synthesis. At the same time, it also activates ULK1 to reduce autophagy as a result. Thus, mTORC1 activation causes phosphorylation of 4EBP, ultimately releasing the sequestration of eIF4E (4EBP is an eIF4E Binding Protein), exposing the phosphorylation sites of eIF4E. The latter has been shown to be indispensable for *Drosophila* growth through increased protein translation^[170]^. Furthermore, the upregulation of *Eif4E* alone recapitulated increased TOR effects on insulin signaling and cardiac aging in flies. On the other hand, *4EBP* overexpression in *Drosophila* helped in protection against cardiac functional aging, promotion of cardiac stress resistance, and maintenance of a normal heart rate^[130]^. In mammalian cells, the overexpression of eIF4E catalyzes fibroblast transformation and increases cell size. However, it is later reversed by increased expression of 4E-BP^[171]^.

The signaling mechanisms of mTORC1 in heart failure are perplexing. Cardiac-specific ablation of mTOR led to dilated cardiomyopathy and early mortality^[172]^, thereby indicating the critical roles of mTOR activity for myocardial growth and proliferation during embryonic and early postnatal stages^[173]^. Inducible ablation of mTOR in adult cardiomyocytes (by tamoxifen induction of adult α-MHC-MerCreMer/Mtor^fl/fl^ mice) also led to fatal dilated cardiomyopathy, thus suggesting the functional significance of mTOR in the maintenance of cardiac physiology and homeostasis^[174]^. These inducible-MHC-mTOR^−/−^ hearts displayed accumulation of 4EBP1, which later inhibited protein translation to cause suppression of cardiomyocytes protein synthesis afterward^[174]^. While these studies support the critical roles of mTOR in cardiac growth, development and homeostasis, moderate mTOR suppression may be beneficial for cardiomyocyte maturation^[175]^. In human inducible pluripotent stem cells (iPS)-derived cardiomyocytes (which are comparable to the fetal stage), mTOR inhibition by Torin 1 shifts cells to a quiescent state and enhances the maturation of these cardiomyocytes. Torin 1 treatment of these iPS-cardiomyocytes decreased p21 and increased p53, cardiac troponin I and K_ir2.1_ cardiac potassium channel, leading to enhanced contractility and maximum oxygen consumption rate^[175]^.

Several components of the mTORC1 pathway have been examined in relation to heart failure. Inducible ablation of Raptor in adult cardiomyocytes by tamoxifen induction of adult α-MHC-MerCreMer/raptor^fl/fl^ aggravated heart failure in response to 1-week of pressure overload. It needs to be noted here that the stated process was completed in the absence of adaptive hypertrophy, consistent with an increase in total and de-phosphorylated *4EBP* (a suppressor of protein translation). These inducible-MHC-Raptor^−/−^ mice also developed spontaneous heart failure with declining ejection fraction (noted 38 days after Raptor ablation), in association with abnormal mitochondria, switch of substrate utilization from fatty acid to glucose oxidation, and increased cardiomyocytes apoptosis^[176]^. These findings suggest the critical role of mTORC1 in cardiac homeostasis and adaptive cardiomyocyte growth in response to pressure overload.

Whereas mTORC1 is essential for cardiac growth and maintenance during development as well as adaptive cardiac hypertrophy in response to stress, excessive mTORC1 activation during chronic stress led to pathological hypertrophy, accumulation of damaged proteins, and heart failure^[177]^. Inhibition of *mTORC1* by RP restores several changes (as mentioned above) and ameliorates heart failure induced by various stressors, including those induced by pressure overload (aortic constriction model)^[178]^, Adriamycin^[179]^, nephrectomy (chronic kidney disease)^[180]^, alcohol^[181]^ or genetic mutations^[182,183]^. The downstream signaling mechanisms of mTORC1 include the regulation of metabolic pathways via mTORC1 target proteins, such as 4EBP1, S6K1 and ULK1. In mice, pressure overload due to transverse aortic constriction activated phosphorylation of ribosomal S6 protein (ribosome biogenesis and protein synthesis) and eukaryotic translation initiation factor-4E (eIF4E; critical for protein translation). The beneficial effect of RP in protecting against pressure-overload-induced heart failure is associated with suppression of *S6K*, phosphorylation of S6, and eIF4E^[184]^, suggesting a potentially critical role of S6K in mediating pathological cardiac hypertrophy. However, cardiac-specific deletion of both S6K1 and S6K2 in mice failed to show a significant effect on the progression of physiological (induced by exercise) or pathological (by pressure overload) cardiac hypertrophy. This implies that the development of cardiac hypertrophy has no critical dependence on S6Ks^[178]^.

The role of 4EBP1, another important downstream target of mTORC1, was tested in our previous study^[185]^. Because mTORC1 inhibition led to suppression of eIF4E and enhancement of 4EBP1, in order to recapitulate the effect of mTORC1 inhibition by RP, we applied pressure-overload heart failure using various genetic mouse models and examined the effect of enhanced 4EBP1^[185]^. The first model: c-4EBP1Tg mice with ~ 9-fold increase in cardiac 4EBP1 protein and mildly decreased cardiac protein translation. These mice exhibited aggravated cardiac systolic dysfunction in both pressure overload and Gαq overexpression-induced heart failure models. The second model: mice with ~ 3-fold increase in a constitutively active mutant 4EBP1 (4EBP1mut, A37/A46), which is resistant to phosphorylation/inactivation by mTOR inhibition, thereby resulting in marked suppression of protein translation. These mice experienced exaggerated heart failure in response to pressure overload (worse than 4EBP1Tg) in the absence of adaptive cardiac hypertrophy (as expected from strong inhibition of protein synthesis). The third model: mice with c-Raptor^+/−^ with decreased but not completely abolished cardiac mTORC1 activity and intact protein translation. These mice ameliorated cardiac systolic function in both pressure overload or Gα q overexpression-induced heart failure models, in parallel with better preservation of cardiac proteome, particularly proteins involved in mitochondrial function, glucose metabolism, and the TCA cycle. Our findings in 3 mouse models suggested that modest inhibition of mTORC1 by RP or c-Raptor^+/−^ is cardioprotective, independent of the effect on protein translation. In contrast, suppression of protein translation by activating 4EBP1 is harmful in the setting of pressure overload or Gαq overexpression-induced heart failure models^[185]^. This is because maintaining protein synthesis is required for adaptive cardiac hypertrophy as a critical compensatory mechanism to cope with cardiac stressors^[186,187]^.

All in all, both hyperactivation and strong suppression of mTORC1 could be deleterious^[173,174,176,188]^. Nevertheless, fine-tuning of mTORC1 in different contexts may be beneficial. An example of fine-tuning of mTORC1 activity is by modulating TSC2, an upstream constitutive inhibitor of mTORC1. The inhibitory effect of TSC2 on mTORC1 is modified by phosphorylation from several kinases, including AMPK and GSK3-β (inhibitors of mTORC1), as well as ERK and Akt (both are stimulators of mTORC1). Research from David Kass’s laboratory identified Serine1365 of TSC2 as a phosphorylation target site of PKG1. Phosphorylation-silencing mutation S1365A-TSC2 aggravated pressure-overload-induced heart failure, due to increased mTORC1 activation that was resistant to PKG1 rescue. In contrast, phosphorylation-mimicking mutation S1365E-TSC2 ameliorated the mentioned heart failure phenotypes. These findings indicate that S1365-TSC phosphorylation is both required and sufficient for the beneficial cardioprotective effect of PKG1 in a pressure overload setting^[189]^. Interestingly, it further showed that mice carrying S1365A-TSC2 knock-in mutations conferred protection against ischemia-reperfusion injury by mTORC1 activation, as the process switched the cardiac substrate utilization from fatty acid oxidation to glycolysis^[190]^.

Rapamycin (RP), as an inhibitor of mTORC1, demonstrates its ability to protect against cardiac aging, as confirmed by multiple laboratories. In a study conducted by Flynn *et al*., late-life RP treatment for three months improved cardiovascular function and attenuated age-associated cardiac inflammation and fibrosis^[142]^. Our studies reported that short-term RP initiated late in life closely resembles the effect of CR in rejuvenating the heart, including regression of age-dependent cardiac hypertrophy, improvement of diastolic function, and preservation of cardiac mitochondrial proteome. It also improves overall protein quality^[97]^. One of the plausible underlying mechanisms of improvement of cardiac protein quality is by enhancing autophagy. RP inhibits ULK phosphorylation and induces autophagy. Interestingly, this process was only observed during the first week of RP treatment as autophagic markers were comparable to baseline levels after two weeks or longer treatment of RP^[159]^. These findings were concomitant with the dynamic changes of mitochondrial biogenesis markers. The transient induction of autophagy and mitochondrial biogenesis by RP suggests that damaged mitochondria were replaced by new mitochondria, thereby helping in the restoration of mitochondrial quality and enhancement of proteostasis^[159]^. The mitochondrial function improvement and overall protein quality may also contribute to the persistent beneficial effect on cardiac function, even following cessation of brief (8 weeks) RP treatment in aged mice from 22 to 24 months old^[191]^.

The “rapamycin memory” discussed above is not limited to cardiac aging. Transient rapamycin treatment for 3 months, initiated for ~20–21 months-old mice, is sufficient to increase life expectancy by ~40%–60% and improve several measures of healthspan in middle-aged mice^[192]^. A recent study reported a long-lasting geroprotection from brief rapamycin treatment in early adulthood^[193]^. This was the result of a persistent increase in intestinal autophagy in *Drosophila.* This “memory” was confirmed by enhanced gut barrier function and Paneth cell architecture in mice exposed to short-term RP treatment^[193]^.

## MTORC2 AND CARDIAC AGING AND HEART FAILURE

Unlike mTORC1, it has been challenging to understand the role of mTORC2 in cardiac function and health, since the specific pharmacological inhibitors targeting mTORC2 are lacking and the upstream activation mechanism of mTORC2 is still not fully understood. Genetic ablations of *Rictor* in mice using cardiomyocyte-specific Cre lead to dilated hearts with systolic dysfunction at 6 months of age^[194]^. The young *Rictor* knock-out mice develop cardiomyopathy shortly after pressure overload, and the hearts become dilated and hypertrophic^[194,195]^. ShRNA-mediated knockdown of *Rictor* increases cardiomyocyte death, pathological remodeling, and cardiac dysfunction upon ischemia damage^[196]^. We recently showed that mTORC2 protects *Drosophila* hearts from high-fat diet-induced cardiomyopathy^[87]^. These studies suggest a cardioprotective effect of mTORC2 in response to various stresses. The protective role of mTORC2 might be due to its regulation of the Hippo pathway, since the phosphorylation of MST1 is elevated in *Rictor* knock-out cardiomyocytes and MST1 inhibition by overexpressing a dominant-negative *MST1* rescues the cardiac dysfunction of *Rictor* knock-out mice^[194]^. In addition, our studies in *Drosophila* demonstrate mitochondrial dynamics as another mechanism for mTORC2-mediated cardiac protection^[87]^.

Besides participating in cell proliferation, cytoskeleton reorganization, and lipid metabolism, mTORC2 is known as a key stress response pathway. *Drosophila* mutants of *Rictor* and *Sin1* exhibit decreased tolerance to heat stress^[197]^. The mTORC2 is activated upon high-fat diet treatment^[87]^ and in response to oxidative stress^[198]^, whereas it is inhibited by ER stress^[199]^. The mTORC2 has also been shown to interact with the Pink1 pathway to regulate mitochondrial homeostasis in *Drosophila* indirect flight muscle^[200]^ and human SHSY5Y neuroblastoma cells^[201]^. These findings suggest that mTORC2 also plays an important role in mitochondria quality control (e.g., mitophagy). Indeed, mTORC2 and AKT signaling have been recently implicated in the regulation of autophagy^[45,202]^. The role of mTORC2 in lifespan regulation is likely attributable to its cytoprotective effects. For example, genetic deletion of *Rictor* decreases lifespan in male mice^[203]^ and cardiac-specific overexpression of R*ictor* prolongs *Drosophila* lifespan^[45]^. However, conflicting results have been found in *C. elegans*^[105,204]^. Different from mTORC1, the effects of mTORC2 in stress resistance and lifespan regulation have not been unequivocally established in multiple organism models and warrant future investigations.

It is well reported that rapamycin-mediated inhibition of mTORC1 attenuates age-related cardiac fibrosis and inflammation, cardiac hypertrophy, and diastolic dysfunction^[97,142]^. However, the role of mTORC2 in cardiac aging is poorly understood. We recently showed that heart-specific overexpression of *Drosophila Rictor* preserves cardiac function and autophagic flux during aging^[45]^, which is consistent with the notion that activation of mTORC2 is cardioprotective and mTORC2 inhibition is detrimental.

## CONCLUSION

The mTOR is a central pathway regulating aging and longevity across multiple model organisms by regulating growth, protein synthesis, lipid metabolism, autophagy, and protein quality control during the aging process. As a sensor kinase, the mTORC1 activity is finely tuned by the levels of intracellular nutrients and energy, growth factors and various cellular stresses. In the heart, the hyperactivation of mTORC1 leads to pathological growth (cardiac hypertrophy) and cardiac dysfunction, as seen in aging and in response to pressure overload stress. Moderate suppression of mTORC1 by calorie restriction, rapamycin or some genetic models of reduced mTORC1 has been shown to ameliorate cardiac aging and pressure-overload-induced heart failure. In contrast, cardiac-specific ablation of mTORC1 leads to dilated cardiomyopathy, as it may suppress protein synthesis required for adaptive cardiac hypertrophy. The role of mTORC2 in cardiac aging is less defined. In contrast to the detrimental effect of mTORC1 activation, overexpression of the mTORC2 component in *Drosophila* is cardioprotective; however, the role of mTORC2 in mammals remains to be elucidated.

## Data Availability

Not applicable.
